# Implication of apoptosis and oxidative stress in mitigation of ivermectin long-term hazards by zinc nanoparticles in male rabbits

**DOI:** 10.1007/s11356-022-24095-1

**Published:** 2022-11-14

**Authors:** Set A. El-Shobokshy, Magda I. Abo-Samaha, Ferial M. Sahwan, Samia M. Abd El-Rheem, Mohamed Emam, Asmaa F. Khafaga

**Affiliations:** 1 Department of Nutrition and Veterinary Clinical Nutrition, Faculty of Veterinary Medicine, Alexandria University, Alexandria, Egypt; 2 Poultry Breeding and Production, Department of Animal Husbandry and Animal Wealth Development, Faculty of Veterinary Medicine, Alexandria University, Alexandria, Egypt; 3 Animal Breeding and Production, Department of Animal Husbandry and Animal Wealth Development, Faculty of Veterinary Medicine, Alexandria University, Alexandria, Egypt; 4 Department of Theriogenology, Faculty of Veterinary Medicine, Alexandria University, Alexandria, Egypt; 5 Department of Nutrition and Veterinary Clinical Nutrition, Faculty of Veterinary Medicine, Damanhour University, Damanhour, Egypt; 6 Department of Pathology, Faculty of Veterinary Medicine, Alexandria University, P. O. Box, Edfina, 22758 Alexandria Egypt

**Keywords:** Ivermectin, Zinc nanoparticles, Caspase, PCNA, Rabbit, Fertility, Growth

## Abstract

Ivermectin is the medication of choice for treating human onchocerciasis and is used in veterinary medicine to treat a variety of ectoparasites and endoparasites. This study was designed to investigate the effects of zinc nanoparticles (ZnNPs) on the fertility of male rabbits exposed to experimental ivermectin (IVM) intoxication. A total of 72 mature male rabbits were equally divided into 4 groups (*n* = 18). The first group (CTR) served as control; the second group (IVM) received subcutaneous injection of IVM (0.2 mg/kg body weight); the third group (ZnNPs) fed on zinc nanoparticles (60 mg/kg diet); and the fourth group (ZnNPs + IVM) were administered IVM and zinc nanoparticles at the same doses. The experiment lasted for 9 weeks. Results revealed that IVM-intoxicated rabbits showed impaired growth performance parameters, including body weight, total body weight gain (TBWG), total feed intake (TFI), and feed conversion ratio (FCR). Moreover, carcass characteristic and fertility parameters (including semen quality parameters and testosterone levels) were also impaired after IVM administration. Additionally, testicular malondialdehyde (MDA) and antioxidant (reduced glutathione, superoxide dismutase, catalase) levels as well as the histopathology and immunohistochemical expression of caspase 3 and PCNA in the testes and epididymis were detrimentally affected. On the contrary, ZnNP administration efficiently improved most of these parameters in IVM-intoxicated rabbits. In conclusion, ZnNPs exhibited promising ability for improving the growth and fertility status of rabbits and reducing the deleterious effects of IVM possibly through the suppression of apoptotic and oxidative pathways.

**Graphical Abstract:**

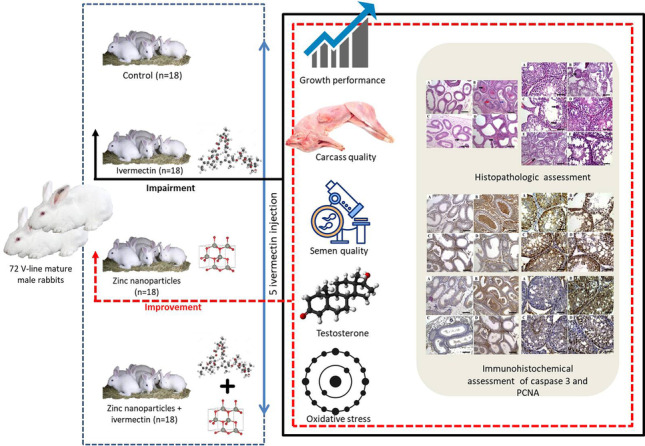

## Introduction

Commercial rabbits have recently gained considerable attention due to their high prolificacy and rapid growth rate compared to broiler chicken (Azazi et al. 2018). Rabbits are an important source of protein for humans because of its high quality and low fat and cholesterol content (PARA et al., 2015). With the increase in rabbit production, the need for animal supplements has become a necessary part of their daily diet. As such, studies into many areas of rabbit production are urgently needed (Abdel-Wareth et al. 2015; Al-Sagheer et al. 2017).

In commercial rabbit production, parasitic infestation, particularly by *Sarcoptes* *scabiei*, which causes mange, has been a major concern (El-Ashram et al. 2020). Ivermectin (IVM) is a drug extensively used to treat and prevent sarcoptic mange, a dangerous condition with serious health implications, including rabbit death (Joshi et al. 2021). This particular drug is an acaricide and anthelmintic developed from avermectin B1, which originates from *Streptomyces avermitilis*. The subcutaneous route has been identified as the most efficient and recommended route of administration, promoting better absorption than the oral and topical routes (Khan Sharun et al. 2019).

IVM is generally well tolerated in mammals (Johnson-Arbor 2022); however, in female rabbits, repeated IVM doses caused pathological alterations in hepatic tissue, including vacuolation of hepatocytes and fibrosis (Al-Jassim et al. 2015). In male rats, therapeutic and double therapeutic doses reduced the total sperm count and induced sperm mortality, as well as pathological abnormalities in the liver, kidneys, and testis. Congestion of blood vessels, degenerative changes (e.g., vacuolar, hydropic, and even necrotic changes), and functional problems of the liver and kidneys are some of the pathogenic changes (Elzoghby et al. 2015). Injecting IVM every 2 weeks has been the standard prophylactic regimen used by rabbit breeders. In addition, IVM is reported to cause excessive generation of reactive oxygen species from the mitochondria, which could potentially interfere with the proper release of cytochrome c, which subsequent activation of caspase-3 and induction of cell apoptosis (Ali et al., 2017). Moreover, it can reduce the expression of proliferating cell nuclear antigen (PCNA), which plays a pivotal role in the S-phase of the cell cycle via DNA synthesis and replication (Strzalka and Ziemienowicz 2011). Therefore, the reduction in PCNA expression may promote suppression of DNA synthesis with subsequent DNA damage (Moshari et al. 2017).

Several metabolic processes and physiological functions of animals rely heavily on trace minerals (Underwood 2012). Zinc (Zn) is the mammalian body’s second most abundant trace element (Dosoky et al. 2022). It cannot be stored in the body and must be consumed on a regular basis to meet physiological requirements (Fairweather-Tait and de Sesmaisons 2019). Zn is an essential component of around 300 enzymes involved in the production and degradation of proteins, lipids, carbohydrates, and nucleic acids, as well as in the metabolism of other micronutrients (Abd El-Hack et al. 2017). It is also a required component of the superoxide dismutase (SOD) enzyme, which plays an important role in the antioxidant defense system (Azad et al. 2017). Additionally, Zn is essential for polynucleotide transcription, which leads to genetic expression, and plays an important role in immune system function, affecting humoral and cellular immunity (Chasapis et al. 2020). Zn boosts T cell production by increasing thymus gland secretion of thymulin. Hence, Zn deficiency results in thymus malfunction, which has a major impact on proper immune function (Mocchegiani et al. 2013). Zn is also required for optimal human physiological activities, such as regular growth and reproduction (Swain et al. 2016).

Recently, several studies have concluded that animals fed zinc nanoparticles (ZnNPs) had better growth, reproduction, and immunity compared to those who were not. To date, however, no available studies have investigated the impact of ZnNPs on the side effects of IVM. Therefore, the present study aimed to determine the possible impact of ZnNPs on the growth and fertility of male rabbits receiving IVM treatment, focusing specifically on the possible mechanisms of action.

## Materials and methods

### Ethical statement

The Institutional Animal Care and Use Committee of University of Alexandria approved the experimental protocol used in this study (Permit #2021/013/97).

### Chemicals

Zn oxide 99.99% (ZnO powder; containing 80.32% Zn, as an inorganic form of Zn) was purchased as a commercial product from Sigma Company, Egypt. Nano-Zn oxide 97% (nZnO powder; containing 77.92% Zn, as a nano-form of Zn) was purchased as a commercial product from Sigma Company, Egypt. The size of Zn oxide nanoparticles was ˂ 50 nm according to the manufacturer company. IVM was purchased from Alfasan International BV Company, the Netherlands.

### Experimental design

A total number of 72 V-line male rabbits (3 months old) were used in this experiment. Rabbits were individually reared in batteries (width × length × height; 44 cm × 50 cm × 35 cm, respectively) of galvanized wire net, equipped with an automatic drinker and a manual feeder. Rabbits were reared in an open house system (naturally ventilated room by windows and ceiling fans). The temperature was adjusted to 19–23 °C. Relative humidity was nearly 60% with a 16-h light and 8-h dark cycle. Fresh tap water was continuously available for consumption via stainless steel nipples located inside each cage. Rabbits were acclimated for 2 weeks before the beginning of experimental procedures.

After acclimatation, animals were divided into four groups (18 bucks/group), and each group was subdivided into three replicates (6 bucks/replicate). Bucks were fed a balanced basal diet containing all the required nutrients according to NRC (1977) (Table 1). The treatment groups were as follows: (1) the first group served as control (CTR) and received a basal diet without ivermectin (IVM) injection; (2) the second group (IVM) received a basal diet with IVM injection; (3) the third group (ZnNPs) received a basal diet with inorganic Zn replacement through ZnNPs (60 mg/kg diet) without IVM injection; and (4) the fourth group (ZnNPs + IVM) received a basal diet with inorganic Zn replacement through ZnNPs (60 mg/kg diet) with IVM injection. IVM was administrated via subcutaneous injection at dose of 0.2 mg/kg body weight (BW). IVM injection was initiated when bucks were 14 weeks old and repeated weekly for five consecutive weeks until 19 weeks old. A pelleted diet was provided ad libitum for animals during the whole experiment.

**Table 1:** The component of basal experimental diet

Ingredients	kg/ton
Corn	50
Barley	150
Wheat bran	250
Oil	30
Molasses	30
B hay	322
SBM 42.9	147
Meth	2
Limestone	2
MCP	9
Salt	5
Mineral premix^1^	1
Vitamin premix^2^	1.5
Anti-mycotoxin	0. 5
Total	1000
Calculated analyses (NRC 1977)	
Digestible energy; kcal/kg	2588.5
Crude protein %	17.05
Ether extract %	5
Crude fiber %	11.97
Starch	15.7

^1^Mineral premix provided per kilogram of diet: manganese, 8.5 mg; iron, 100 mg; copper, 10 mg; cobalt, 0.3 mg; iodine, 0.2 mg; selenium, 0.3 mg; zinc, 60 mg. Each 1-kg mineral premix contains the following: Mn sulfate (34.55 g), iron carbonate (207.47 g), copper oxide (12.52 g), cobalt oxide (0.42 g), pot iodide (0.26 g), sodium selenite (0.657 g), Zn oxide (74.63 g), and carrier (limestone) up to 1 kg

^2^Each 1 kg of vitamins premix contained the following: vitamin A, 10,000 IU; vitamin D_3_, 1800 UI; vitamin E, 15 mg; vitamin K_3_, 4.5 mg; vitamin B_1_, 0.5 mg; vitamin B_2_, 4 mg; vitamin B_12_, 0.001 mg; folic acid, 0.1 mg; pantothenic acid, 7 mg; nicotinic acid, 20 mg

### Growth performance parameters

Individual live BWs were recorded as the initial BW at 12 weeks old and every 2 weeks until the end of the experiment (the final BW was obtained at 21 weeks old). Total weight gain and total feed intake were recorded, after which the average feed conversion ratio, that is, the amount of total feed intake/total body gain, was calculated.

### Assessments of carcass characteristics

For the assessment of carcass characteristics, nine rabbits from each treatment were randomly selected. Rabbits were weighed in a fasted state before slaughtering to determine the live body weight. After the slaughtered rabbits were bled, the skin, genitals, urinary bladder, gastrointestinal tract, and the distal part of the legs were removed. Hot carcasses (with the head, thoracic cage organs, liver, kidneys, and perirenal and scapular fat) were weighed. The dressing percentage and the ratio of thigh, skin (with head skin), skin (without head skin), head with skin, head without skin, heart, lung, liver, spleen, abdominal fat, and kidney relative to the live BW were calculated.

### Dimensions and relative weight of the reproductive organs

Organ dimensions (scrotum, penile length, and testicular circumference, length, and width) were measured after slaughter. Circumference was measured using a measuring tape, whereas testicular length was measured using an obstetrical pelvimeter.

### Epididymal sperm preparation

Immediately after slaughtering, the animals were dissected and the epididymis was collected as quickly as possible and placed in a clean Petri plate. Thereafter, the cauda epididymis was separated from the whole epididymis, cut into several pieces, immersed in 3 mL pre-warmed phosphate buffer saline (PBS) solution, and incubated for 10 min at 37 °C to allow for sperm release from the epididymal lumen (Mangoli et al. 2013). The sperm count was evaluated using a hemocytometer chamber (count × 10^6^) at 40 × magnification using light microscopy (Olympus Co., Tokyo, Japan). The percentages of progressive motility and viability were evaluated for at least 200 spermatozoa from each buck. Assessment of motile sperm at the warm stage showed progressive forward movement under 100 × magnification using a light microscope. Assessment of live and dead sperm was performed by counting 200 sperm cells using an eosin-nigrosin staining mixture. Complete or partial purple-stained sperm cells were considered non-viable, whereas non-stained sperm cells were considered viable.

### Plasma testosterone measurement

Using an indirect enzyme immunoassay assay kit (Monobind, 100 North point Drive, Lake Forest, CA), plasma testosterone levels were estimated following the methods described by Tietz (1995).

### Antioxidant indicators

At the end of the experimental period, rabbits were slaughtered and dissected. The testes were carefully removed, cut into small pieces, and preserved at − 20 °C for further analysis of Zn, reduced glutathione (GSH), superoxide dismutase (SOD), catalase (CAT), and malondialdehyde (MDA). Testicular Zn (CAT. Zn 21 20, Biodiagnostic), SOD (CAT. SD 25 21, Biodiagnostic), and CAT (Cat. CA 2517, Biodiagnostic) were assessed using spectrophotometric procedures (Hitachi spectrophotometer, Tokyo, Japan) with commercially available kits (Biodiagnostic Co., Dokki, Giza, Egypt) according to the manufacturer’s instructions. GSH (CAT. No. GR 25 11, Biodiagnostic) and MDA (Cat. MD 2529, Biodiagnostic) testicular contents were examined using the colorimetric method according to Beutler et al. (1963) and Okhawa et al. (1979), respectively.

### Histopathological study

Immediately after slaughtering, specimens were collected from testes of control and treated bucks. The collected samples were washed and immersed for 48 h in a 10% neutral-buffered formalin solution for fixation. Fixed samples were prepared using the routine paraffin-embedding technique (Bancroft 2013). Briefly, fixed samples were dehydrated in ascending grades of ethanol, cleared using several changes of xylene, prepared in paraffin blocks, and microtomed into 3–5-µm-thick sections. Prepared sections were routinely stained with hematoxylin and eosin (H & E staining). Thereafter, blinded examination and image capture were performed by an experienced pathologist. Representative photomicrographs were obtained using a digital camera (Leica EC3; Leica, Germany) connected to a microscope (Leica DM500). Scoring for spermatogenesis was performed via Johnsen’s scoring system. The presence or absence of the primary cell types and/or lesions was scored from 1 to 10 according to Johnsen (1970) and Hassan et al. (2019). Twenty seminiferous tubules were randomly selected in each cross-section and scored under a light microscope (× 400); the mean score was determined for each group.

### Immunohistochemical study

For immunohistochemical evaluation, each paraffin block was cut into several 4-µm-thick sections and rehydrated in decreasing concentrations of ethanol. Antigen retrieval was done in citrate-buffered saline (0.01 mol/L, pH 6.0), and endogenous peroxidase activity was quenched in phosphate-buffered saline with H_2_O_2_ 0.3% (v/v) (PBS). Thereafter, sections were incubated for 1 h with 10% (v/v) normal goat serum to block non-specific immunologic reagent binding, and tissue sections were incubated overnight at 4 °C with anti-Caspase-3 antibody rabbit monoclonal [EPR18297] (Cat, ab184787, Abcam, Cambridge, UK) and Anti-PCNA Mouse monoclonal antibody [24/PCNA] (Cat, ab280088 Abcam, Cambridge, UK). Afterwards, sections were washed in PBS and treated for 60 min with biotin-conjugated goat anti-rabbit IgG antiserum (Histofine kit, Nichirei Corporation, Japan). The sections were then rewashed in PBS and treated for 30 min with streptavidin-peroxidase conjugate (Histofine kit, Nichirei Corporation, Japan). The streptavidin–biotin complex was observed for 3 min with a 3,3′-diaminobenzidine tetrahydrochloride (DAB)-H_2_O_2_ solution. Finally, Mayer’s hematoxylin solution was used to counterstain the sections. Several original micrographs were obtained from five high-power fields/sections/organs at random and used for quantitative histomorphometric examination of immunostaining. Caspase-3 and PCNA positive brown color cells were counted in each micrograph (HPF, × 40) using manual computer-assisted cell counting (ImageJ plug-in-cell counter.jar) with ImageJ (v1.46 r, NIH, Bethesda, MD, USA) (Schneider et al. 2012) as reported by Powell et al. (2014). For each group, the mean count of immunological positive cells was computed and analyzed using non-parametric statistics (Saleh et al. 2020).

### Statistical analyses

Statistical analyses of the data were performed using SAS software (SAS 2014). One-way analysis of variance (ANOVA) was used for data analysis. Duncan’s test was used when treatment effects were significant. The overall significance level was set at *P* < 0.05. All values were expressed as mean ± standard error. Histomorphometric analysis of caspase-3 and PCNA immune expressions were analyzed using the non-parametric analysis using Kruskal–Wallis test to assess the significance between mean scores obtained from Wilcoxon rank-sum test.

## Results

### Growth performance

The effects of dietary inorganic Zn replacement through ZnNPs with or without IVM injection on the growth performance of buck rabbits are presented in Table 2. The statistical analysis of our data revealed no significant differences between the live BW of different experimental groups at baseline and 2 weeks later (12 and 14 weeks old). However, rabbits in the IVM and IVM + ZnNPs groups showed a significant (*P* < 0.0001) reduction in live BW at 18, 20, and 21 weeks old compared to the control group. In addition, IVM-treated rabbits had significantly lower total BW gain (TBWG) and total feed intake (TFI) (*P* < 0.0001) compared to control group, which caused worst FCR. Conversely, rabbits in the ZnNPs group had significantly higher TFI (*P* < 0.0001) compared to control rabbits. Interestingly, rabbits in the ZnNPs + IVM group showed significant greater improvement in live BW at 16, 18, and 20 weeks old and TFI, with a subsequent improvement in FCR, compared to the IVM group.

**Table 2:** Effects of dietary supplementation of inorganic zinc or zinc nanoparticles with or without ivermectin (IVM) treatment on growth performance of rabbit males

Variable	Group				*P* value
	CTR	IVM	ZnNPs	ZnNPs + IVM	
Initial bwt (kg) (12 weeks)	1.24 ± 0.05	1.22 ± 0.02	1.24 ± 0.03	1.22 ± 0.09	NS
14 weeks (kg)	1.85 ± 0.07	1.89 ± 0.03	1.80 ± 0.03	1.91 ± 0.03	NS
16 weeks (kg)	2.25 ± 0.00^a^	2.08 ± 0.01^b^	2.26 ± 0.06^a^	2.26 ± 0.04^a^	0.0066
18 weeks (kg)	2.51 ± 0.01^a^	2.19 ± 0.02^c^	2.51 ± 0.07^a^	2.37 ± 0.04^b^	< .0001
20 weeks (kg)	2.70 ± 0.01^a^	2.34 ± 0.02^c^	2.71 ± 0.06^a^	2.52 ± 0.04^b^	< .0001
Final bwt (kg) (21 weeks)	2.77 ± 0.02^a^	2.46 ± 0.00^b^	2.89 ± 0.10^a^	2.60 ± 0.04^b^	< .0001
TBWG (kg)	1.53 ± 0.07^ab^	1.23 ± 0.03^c^	1.65 ± 0.06^a^	1.38 ± 0.03^bc^	< .0001
TFI (kg)	7.01 ± 0.09^b^	6.11 ± 0.44^c^	7.40 ± 0.06^a^	6.67 ± 0.11^b^	< .0001
AFCR (kg)	4.58 ± 0.20	4.95 ± 0.06	4.48 ± 0.135	4.83 ± 0.06	NS

The data presented as mean ± standard error. Means bearing different superscript letters within the same row are significantly different (*P* < 0.05)

*NS* non-significant, *TG* total body weight gain, *TFI* total feed intake, *AFCR* average feed conversion ratio

### Carcass quality

The effects of dietary ZnNP supplementation with or without IVM injection on dressing percentage and carcass quality of buck rabbits are shown in Table 3. Tabulated results revealed that the dressing percentage was significantly increased (*P* < 0.0001) in the ZnNPs group and significantly lower (*P* < 0.0001) in the IVM group compared to the control group. However, the relative weight of the thigh was significantly improved (*P* < 0.0001) in the ZnNPs group compared to the control group. Conversely, skin (with or without head skin) and abdominal fat showed no significant change (*P* < 0.0001) compared to the control group. In addition, the relative weights of the head with skin, heart, and lungs were significantly lower (*P* < 0.0001) compared to the control group. The relative weights of the head without skin and spleen were significantly decreased in IVM group (*P* < 0.0001) compared to the control group. Additionally, the relative weights of the liver were significantly greater (*P* < 0.0001) in the IVM group and significantly lower (*P* < 0.0001) in ZnNPs and ZnNPs + IVM groups compared to control group. The relative weight of the kidney was significantly lower (*P* < 0.0001) in the IVM and ZnNPs + IVM groups compared to the control group.

**Table 3:** Effects of dietary supplementation of inorganic zinc or zinc nanoparticles with or without ivermectin (IVM) treatment on dressing percentages of rabbit males

Variable	Group				*P* value
	CTR	IVM	ZnNPs	ZnNPs + IVM	
Live body wt. (g)	2536.22 ± 17.68^c^	2508.00 ± 12.12^c^	2635.00 ± 10.10^b^	2793.89 ± 32.54^a^	< .0001
Dressing (%)	52.39 ± 0.20^b^	51.02 ± 0.45^c^	53.71 ± 0.36^a^	53.36 ± 0.28^ab^	< .0001
Thigh (%)	21.24 ± 0.32^b^	21.59 ± 0.05^b^	22.43 ± 0.02^a^	21.24 ± 0.13^b^	0.0022
Skin (with head skin) (%)	16.30 ± 0.61	16.42 ± 0.13	16.12 ± 0.28	16.02 ± 0.26	NS
Skin (without head skin) (%)	12.76 ± 0.63	12.87 ± 0.15	13.52 ± 0.24	12.90 ± 0.30	NS
Head with skin (%)	19.34 ± 0.21^a^	18.53 ± 0.03^b^	16.88 ± 0.09^d^	18.01 ± 0.01^c^	< .0001
Head without skin (%)	6.63 ± 0.15^a^	6.14 ± 0.09^b^	6.43 ± 0.08^a^	6.50 ± 0.05^a^	0.0105
Heart (%)	0.39 ± 0.01^a^	0.34 ± 0.02^b^	0.28 ± 0.00^c^	0.30 ± 0.01^c^	< .0001
Lung (%)	0.64 ± 0.05^a^	0.54 ± 0.01^b^	0.42 ± 0.00^c^	0.46 ± 0.02^c^	< .0001
Liver (%)	2.58 ± 0.08^b^	2.87 ± 0.03^a^	2.33 ± 0.01^c^	2.41 ± 0.10b^c^	< .0001
Spleen (%)	0.06 ± 0.005^a^	0.04 ± 0.00^b^	0.06 ± 0.00^a^	0.05 ± 0.00^a^	0.0049
Abdominal fat (%)	0.10 ± 0.107	1.03 ± 0.122	1.06 ± 0.11	1.00 ± 0.17	NS
Kidney (%)	0.66 ± 0.02^a^	0.56 ± 0.01^b^	0.51 ± 0.01^c^	0.62 ± 0.01^a^	< .0001

The data presented as mean ± standard error. Means bearing different superscript letters within the same row are significantly different (*P* < 0.05)

*NS* non-significant

### Dimensions and relative weight of reproductive organs

Data in Table 4 shows that the diameter of the scrotum was significantly lower in the IVM group and significantly greater (*P* < 0.0001) in ZnNPs group compared to the control group. However, the penile length of the different groups differed significantly (*P* < 0.0001) compared to that of the control group, with ZnNPs group and CTR group having the greatest and least values (1.95 and 1.50 cm, respectively). In addition, testicular circumference and length were significantly lower (*P* < 0.0001) in all treatment groups compared to the control group. However, testicular width was significantly higher (*P* < 0.0001) in ZnNPs + IVM compared to control group.

**Table 4:** Effects of dietary supplementation of inorganic zinc or zinc nanoparticles with or without ivermectin (IVM) treatment on organ size, sperm motility, viability, and concentration of rabbit males

Variable	Group				*P* value
	CTR	IVM	ZnNPs	ZnNPs + IVM	
Organ dimensions					
Scrotum (cm)	5.45 ± 0.01b^b^	5.33 ± 0.08^c^	5.70 ± 0.06^a^	5.63 ± 0.12^ab^	0.008
Penis length (cm)	1.50 ± 0.00^c^	1.77 ± 0.07^b^	1.95 ± 0.01^a^	1.90 ± 0.08^ab^	< .0001
Testis circumference (cm)	4.25 ± 0.07^a^	3.63 ± 0.08^c^	3.85 ± 0.04^b^	4.03 ± 0.06^b^	< .0001
Testis length (cm)	4.05 ± 0.04^a^	3.30 ± 0.16^b^	3.10 ± 0.12^b^	3.03 ± 0.07^b^	< .0001
Testis width (cm)	1.20 ± 0.00^b^	1.23 ± 0.02^b^	1.35 ± 0.04^b^	1.67 ± 0.09^a^	< .0001
Relative organ weight					
Testis (%)	0.10 ± 0.00	0.10 ± 0.01	0.10 ± 0.00	0.10 ± 0.00	NS
Epididymis (%)	0.09 ± 0.00	0.09 ± 0.00	0.10 ± 0.00	0.09 ± 0.00	NS
Pituitary gland (%)	0.01 ± 0.00	0.01 ± 0.00	0.01 ± 0.00	0.01 ± 0.00	NS
Accessory gland (%)	0.16 ± 0.01^b^	0.18 ± 0.01^b^	0.25 ± 0.02^a^	0.22 ± 0.01^a^	< .0001
Semen quality parameters					
Sperm motility (%)	51.67 ± 3.63^b^	50.00 ± 5.77^b^	83.33 ± 3.33^a^	60.00 ± 2.89^b^	< .0001
Sperm livability (%)	62.33 ± 6.50^b^	60.33 ± 5.05^b^	87.00 ± 3.00^a^	67.67 ± 3.61^b^	0.0013
Sperm concentration (× 10^6^/ml)	583.33 ± 58.33^b^	325.00 ± 50.52^c^	733.33 ± 8.33^a^	675.00 ± 7.22^ab^	< .0001

The data presented as mean ± standard error. Means bearing different superscript letters within the same row are significantly different (*P* < 0.05)

Conversely, the weight of the testis, epididymis, and pituitary gland did not differ significantly among groups. However, the weight of the accessory glands was significantly increased (*P* < 0.0001) in ZnNPs and ZnNPs + IVM group compared to the control group (Table 4).

### Semen quality parameters

As shown in Table 4, sperm motility and livability were significantly higher in ZnNPs group compared to the control group. However, sperm concentrations were significantly lower in the IVM and ZnNPs compared to the control group.

### Testosterone concentration and age at puberty

In Table 5, serum testosterone concentrations at 16 weeks old were significantly (*P* < 0.0026) higher in ZnNPs group compared to the control group. Meanwhile, serum testosterone concentration at 18 weeks old was significantly higher (*P* < 0.0001) in ZnNPs and ZnNPs + IVM groups than in the control group. At 20 weeks old, serum testosterone concentrations did not significantly differ (*P* < 0.0001) among the groups.

**Table 5:** Effects of dietary supplementation of inorganic zinc or zinc nanoparticles with or without ivermectin (IVM) treatment on testosterone hormone level (pg/ml) in rabbit males

Variable	Group				*P* value
	CTR	IVM	ZnNPs	ZnNPs + IVM	
Testosterone (pg/ml) at 16 weeks	0.62 ± 0.10^b^	0.55 ± 0.06^b^	2.09 ± 0.44^a^	0.94 ± 0.10^b^	0.0026
Testosterone (pg/ml) at 18 weeks	0.13 ± 0.02^b^	0.97 ± 0.04^b^	4.33 ± 1.34^a^	3.85 ± 1.08^a^	0.0001
Testosterone (pg/ml) at 20 weeks	2.29 ± 0.40	1.98 ± 0.16	2.94 ± 0.37	2.65 ± 0.74	NS
Age at puberty (weeks)	19 ± 0.29^ab^	20 ± 0.00^a^	18 ± 0.58^b^	18 ± 0.58^b^	0.0066

The data presented as mean ± standard error. Means bearing different superscript letters within the same row are significantly different (*P* < 0.05)

*NS* non-significant

Additionally, the age at puberty was recorded for all groups; ZnNPs and Zn-NPs + IVM groups reached puberty (around 18 weeks of age) significantly earlier (*P* < 0.0066) than the other groups. Meanwhile, the CTR and IVM groups reached puberty at 19 and 20 weeks old, respectively (Table 5).

### Antioxidant indicators

As shown in Table 6, dietary replacement of inorganic Zn with ZnNPs significantly enhanced testicular Zn concentrations as compared to control. The CAT enzyme was significantly lower in the IVM group compared to the control group. In contrast, GSH concentrations were significantly greater in the ZnNPs group compared to the control group. Interestingly, SOD concentrations were significantly higher in the ZnNPs and ZnNPs + IVM groups compared to the control group. Meanwhile, the same marker (SOD) was significantly lower in the IVM group compared to the control group.

**Table 6:** Effects of dietary supplementation of inorganic zinc or zinc nanoparticles with or without ivermectin (IVM) treatment on zinc level in testicles and antioxidants in testicles of rabbit males

Variable	Group				*P* value
	CTR	IVM	ZnNPs	ZnNPs + IVM	
Zn (µg/g)	2.03 ± 0.05^b^	1.75 ± 0.36^b^	2.90 ± 0.14^a^	2.05 ± 0.03^b^	0.0017
CAT (µg/g tiss)	41.50 ± 1.01^a^	18.33 ± 0.88^b^	45.33 ± 1.69^a^	44.00 ± 1.44^a^	< .0001
GSH (mg/dl)	32.33 ± 6.09^bc^	23.33 ± 1.36^c^	45.50 ± 0.43^a^	38.50 ± 1.01^ab^	0.0002
SOD (µg/g tiss)	50.00 ± 0.29^b^	28.33 ± 1.30^c^	63.67 ± 1.64^a^	62.00 ± 1.73^a^	< .0001
MDA (Nmol/g tiss)	3.65 ± 0.13^b^	9.27 ± 0.29^a^	2.87 ± 0.07^c^	3.40 ± 0.06^b^	< .0001

The data presented as mean ± standard error. Means bearing different superscript letters within the same row are significantly different (*P* < 0.05)

Our findings showed that the level of testicular MDA (an index of oxidative process) was significantly improved in the IVM group and significantly lower in ZnNPs group compared to the control group. Meanwhile, rabbits in ZnNPs + IVM group showed no significant difference in MDA levels compared to the control group.

### Histopathologic findings

**Fig. 1:**
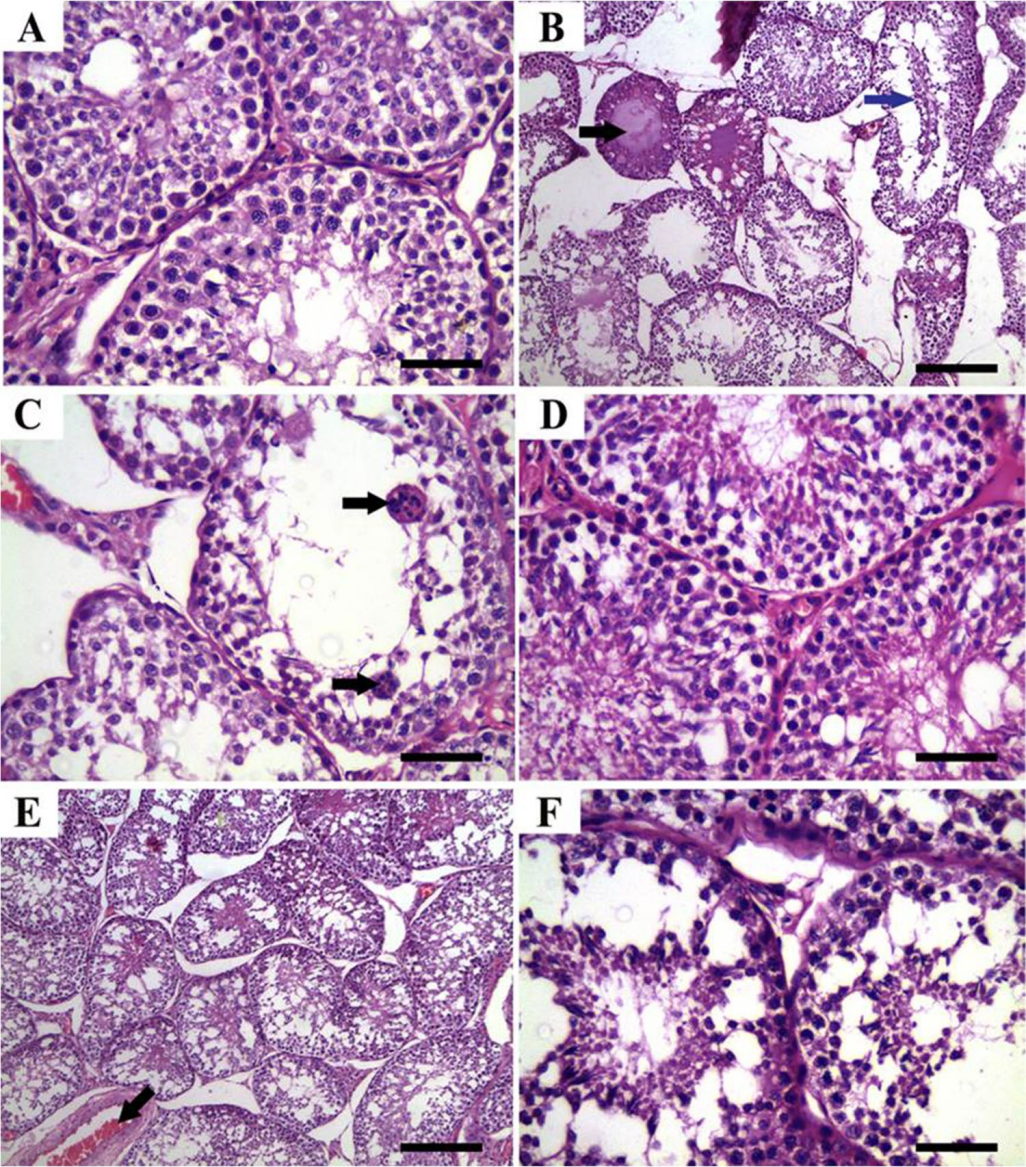
Representative photomicrograph of testicular tissues of rabbits from control (**A**), IVM (**B**, **C**), ZnNPs (**D**), and ZnNPs + IVM (**E**, **F**) showed normal histoarchitecture of seminiferous tubules, interstitial tissues, spermatogenic cells, Sertoli cells, and Leydig cells (**A**, **D**). Abnormal arrangement and vacuolization of spermatogenic cells, eosinophilic proteinaceous materials (black arrow) and necrotic debris (blue arrow) occupying the central regions of the tubular laminae (**B**), formation of sperm giant cells (arrows) (**C**), and mild to moderate vacuolization and degeneration of spermatogenic cells and intertubular congestion (arrow) (**E**, **F**). H&E, *scale bar* = 50 µm (**A**, **C**, **D**, **F**), *scale bar* = 200 µm (**B**, **E**)

As shown in Fig. 1, testicular tissues of rabbits from the CTR and ZnNPs group showed normal histoarchitecture of seminiferous tubules, interstitial tissues, spermatogenic cells, Sertoli cells, and Leydig cells (Fig. 1A, D). In contrast, those that received IVM (IVM group) showed abnormal arrangement, degeneration, and vacuolization of spermatogenic cells. Moreover, necrosis of the epithelial cell lining the seminiferous tubules and multifocal separation of basement membrane were observed. Furthermore, necrotic spermatocytes and/or eosinophilic proteinaceous materials were identified with cellular debris occupying the central regions of the tubular laminae (Fig. 1B) and characteristic formation of a high number of sperm giant cells (Fig. 1C). Moreover, Sertoli cells were apparently reduced, whereas the intertubular tissues were widened and contained sparse distribution of Leydig cells with congested intertubular blood vessels. These lesions were ameliorated after inorganic Zn replacement through Zn nanoparticles in ZnNPs + IVM group (Fig. 1E, F).

**Fig. 2:**
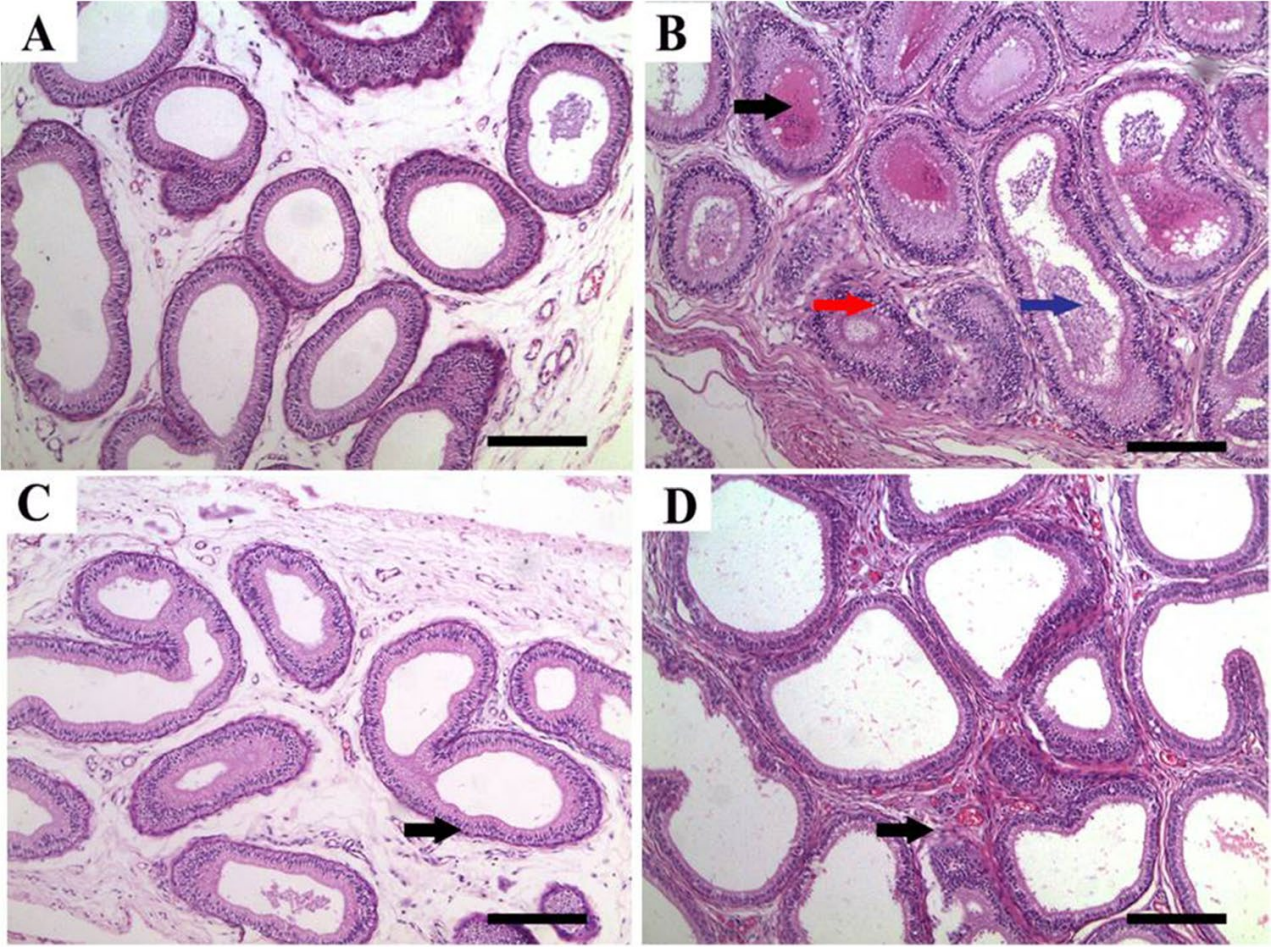
Representative photomicrograph of epididymal tissues of rabbits from control (**A**), IVM (**B**), ZnNPs (**C**), and ZnNPs + IVM (**D**) showed normal histologic limits of epididymal tubules (**A**, **C**), necrotic eosinophilic proteinaceous materials (black arrow) and cellular debris (blue arrow) occupying the central regions of the tubular laminae, degeneration and necrosis in epithelial lining (red arrow) (**B**), and amelioration of the most of these lesions with interstitial fibrosis and congestion of intertubular capillaries (arrow) (**D**). H&E, *scale bar* = 50 µm (**A**, **C**, **D**), *scale bar* = 200 µm (**B**)

**Fig. 3:**
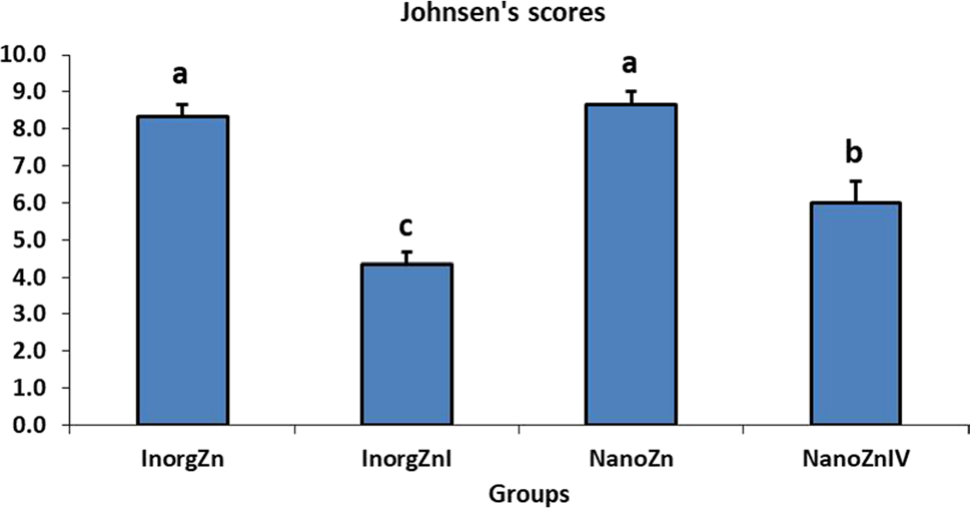
Ameliorating effect of oral administration of ZnNPs (60 mg/kg diet) on spermatogenesis score of male rats intoxicated with IVM (0.2 mg/kg bwt) for 5 weeks. Data are expressed as the mean ± SEM. Different letters are significant at *P* < 0.05 with respect to the control group as a negative control (ANOVA with Dunnett’s multiple comparison test)

Epididymal tissues showed normal histologic limits of the epididymal tubules in the CTR and ZnNPs groups (Fig. 2A, C). Meanwhile, tissues from the IVM group showed necrotic eosinophilic proteinaceous materials and cellular debris occupying the central regions of the tubular laminae, degeneration and necrosis of epididymal tubule epithelial cells, and interstitial tissue fibrosis (Fig. 2B). Conversely, tissues from ZnNPs + IVM group exhibited amelioration of the most of these lesions. Interstitial tissue fibrosis and intertubular capillary congestion were evident in this group (Fig. 2D). The scoring of spermatogenesis was significantly (*P* < 0.05) declined in IVM group compared to control rats. However, oral administration of ZnNPs in ZnNPs + IVM group significantly (*P* < 0.05) improved the score of spermatogenesis compared to rats in IVM group (Fig. 3).

### Immunohistochemical findings

**Fig. 4:**
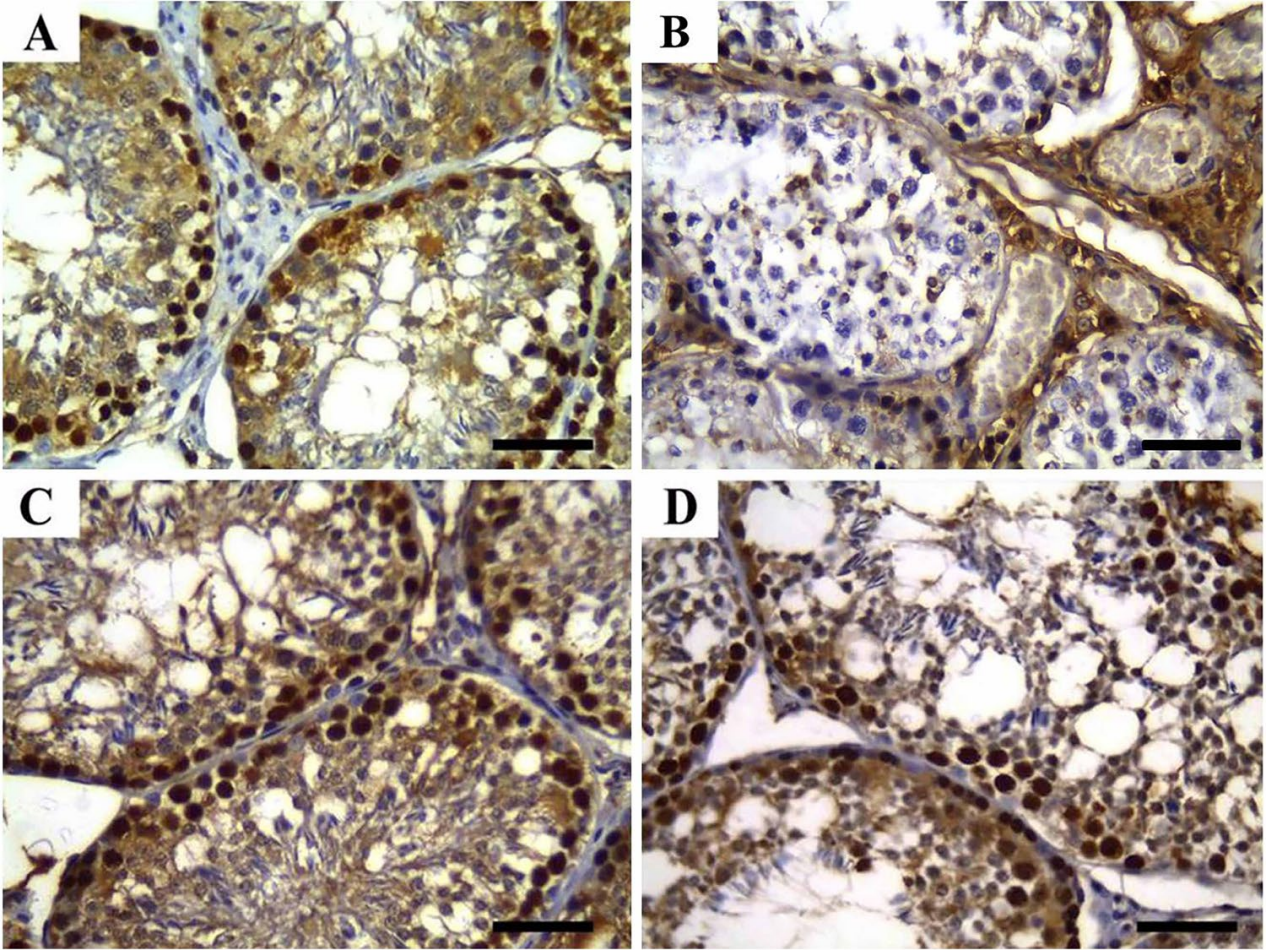
Representative photomicrograph of testicular tissues of rabbits from control (**A**), IVM (**B**), ZnNPs (**C**), and ZnNPs + IVM (**D**) (sections stained with anti-PCNA immune staining; scale bar = 50 μm) showed strong (**A**, **C**, **D**) and moderate immunoreactivity of PCNA in testicular spermatogenic cells

**Fig. 5:**
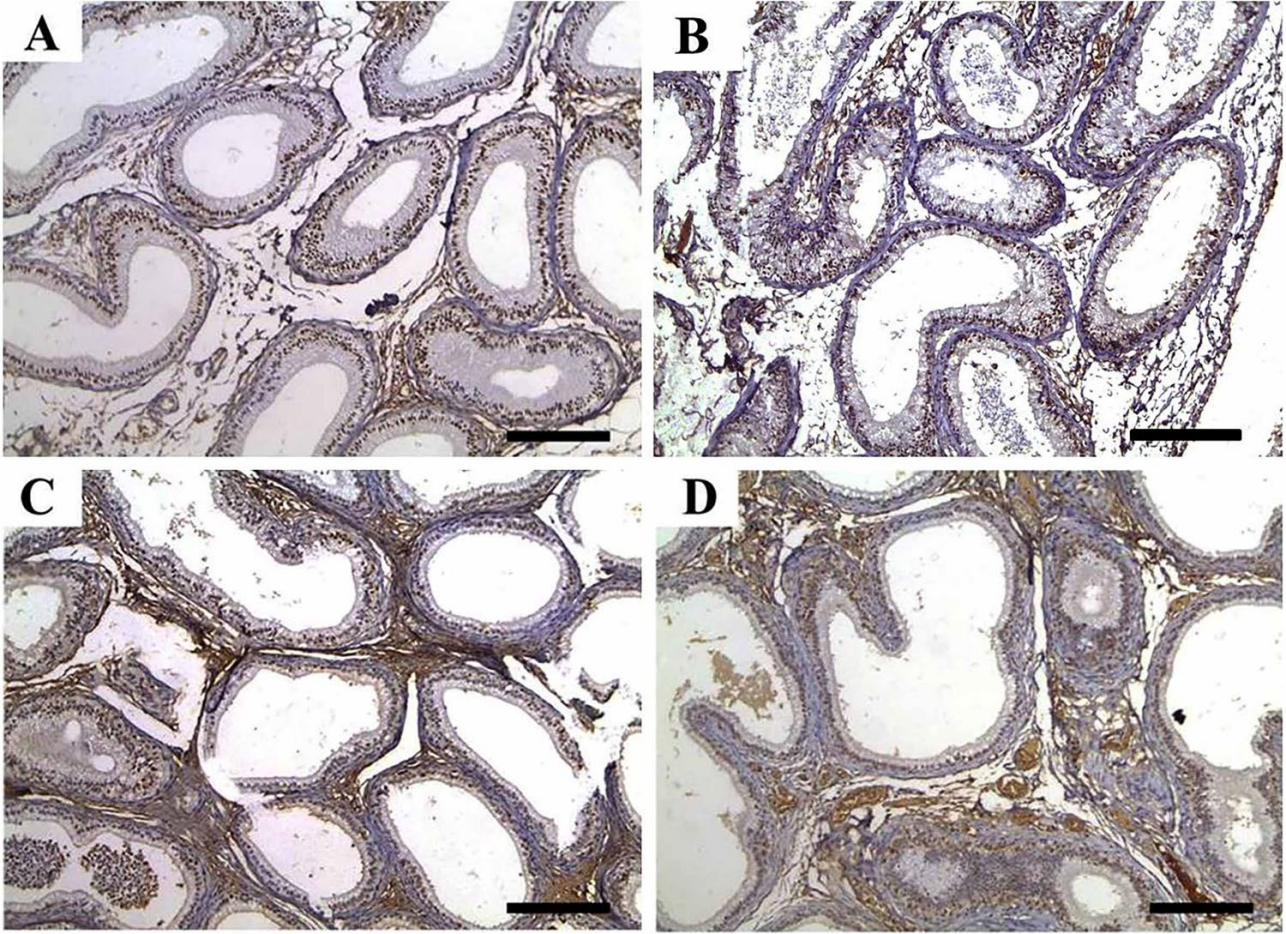
Representative photomicrograph of testicular tissues of rabbits from control (**A**), IVM (**B**), ZnNPs (**C**), and ZnNPs + IVM (**D**) (sections stained with anti-PCNA immune staining; scale bar = 50 μm) showed strong (**A**, **C**) and mild to moderate immunoreactivity of anti-PCNA in lining epithelium of epididymis and strong immunoreactivity of intraluminal debris and interstitium (**B**)

Apoptotic activity of testicular spermatogenic cells and columnar epithelium of epididymal tubules were evaluated via immunohistochemical localization of caspase-3 (Figs. 4 and 5). In the testes, the immunoreactivity of caspase-3 was greatest in the IVM group followed by ZnNPs + IVM compared to CTR and ZnNPs groups. The highest apoptotic activity of the testicular cells was detected in the IVM group. In the epididymis, the immune staining of caspase-3 was significantly greatest in the IVM group followed by ZnNPs + IVM group compared to the CTR and ZnNPs groups. Figure 7 shows the non-parametric analysis for the mean count of immunological positive cells in the testes and epididymis.

**Fig. 6:**
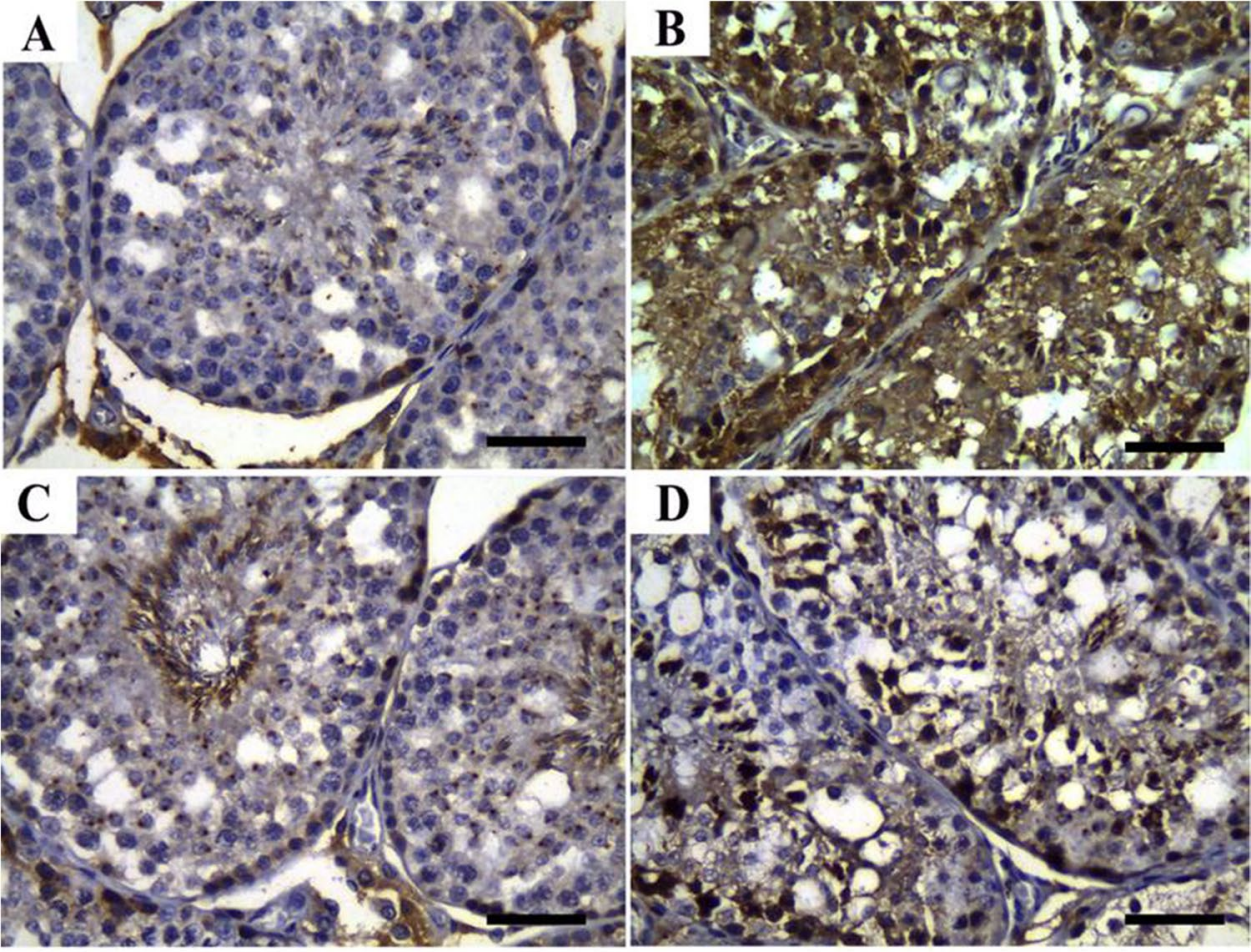
Representative photomicrograph of testicular tissues of rabbits from control (**A**), IVM (**B**), ZnNPs (**C**), and ZnNPs + IVM (**D**) (sections stained with anti-caspase-3 immune staining; scale bar = 50 μm) showed strong (**B**), moderate (**D**), and mild to negative immunoreactivity of anti-caspase-3 in testicular spermatogenic cells

**Fig. 7:**
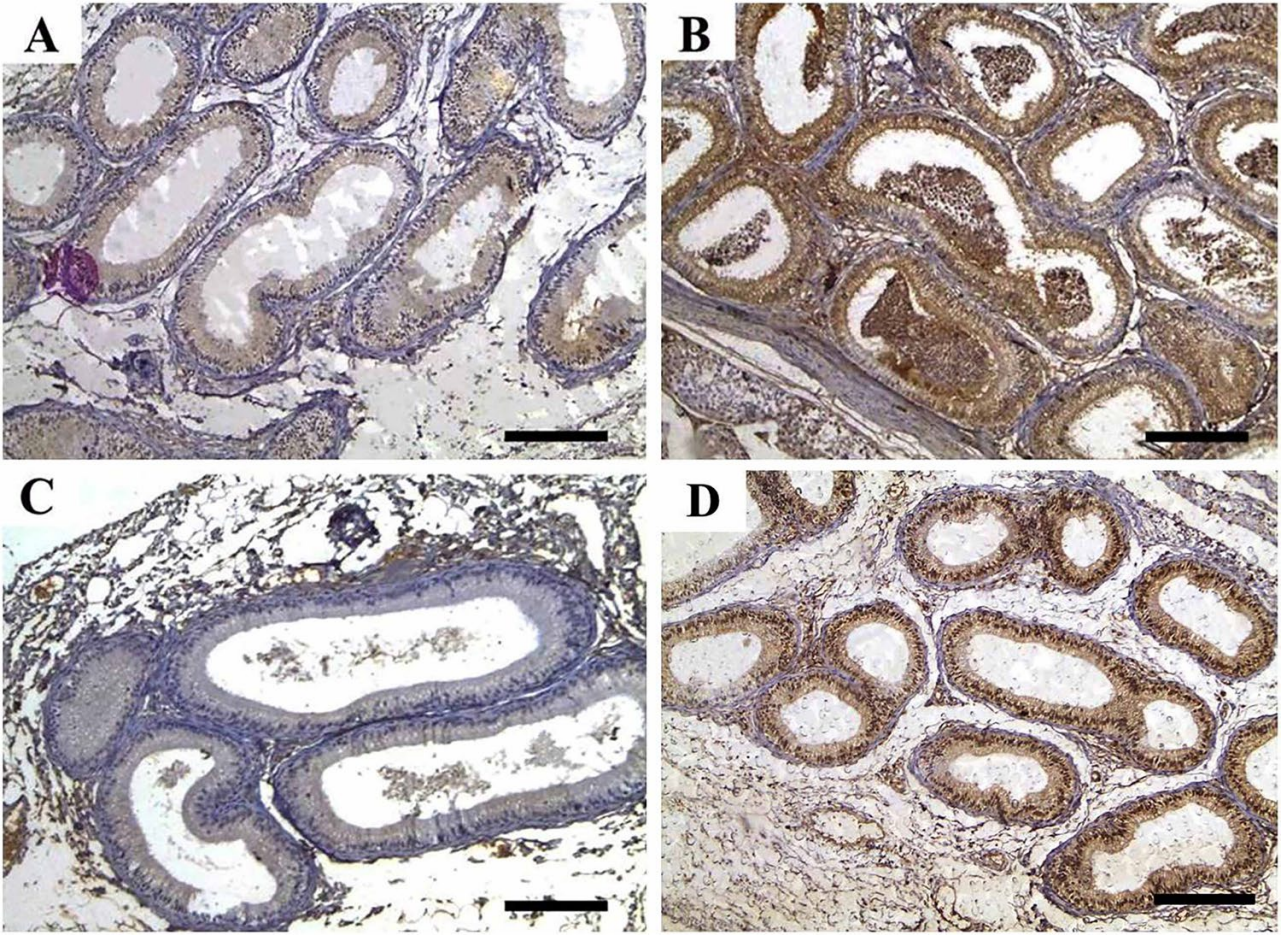
Representative photomicrograph of testicular tissues of rabbits from control (**A**), IVM (**B**), ZnNPs (**C**), and ZnNPs + IVM (**D**) (sections stained with anti-caspase-3 immune staining; scale bar = 50 μm) showed strong (**B**), moderate (**D**), and negative immunoreactivity of anti-caspase-3 in lining epithelium of epididymis and strong immunoreactivity of intraluminal debris and interstitium (**B**, **D**)

**Fig. 8:**
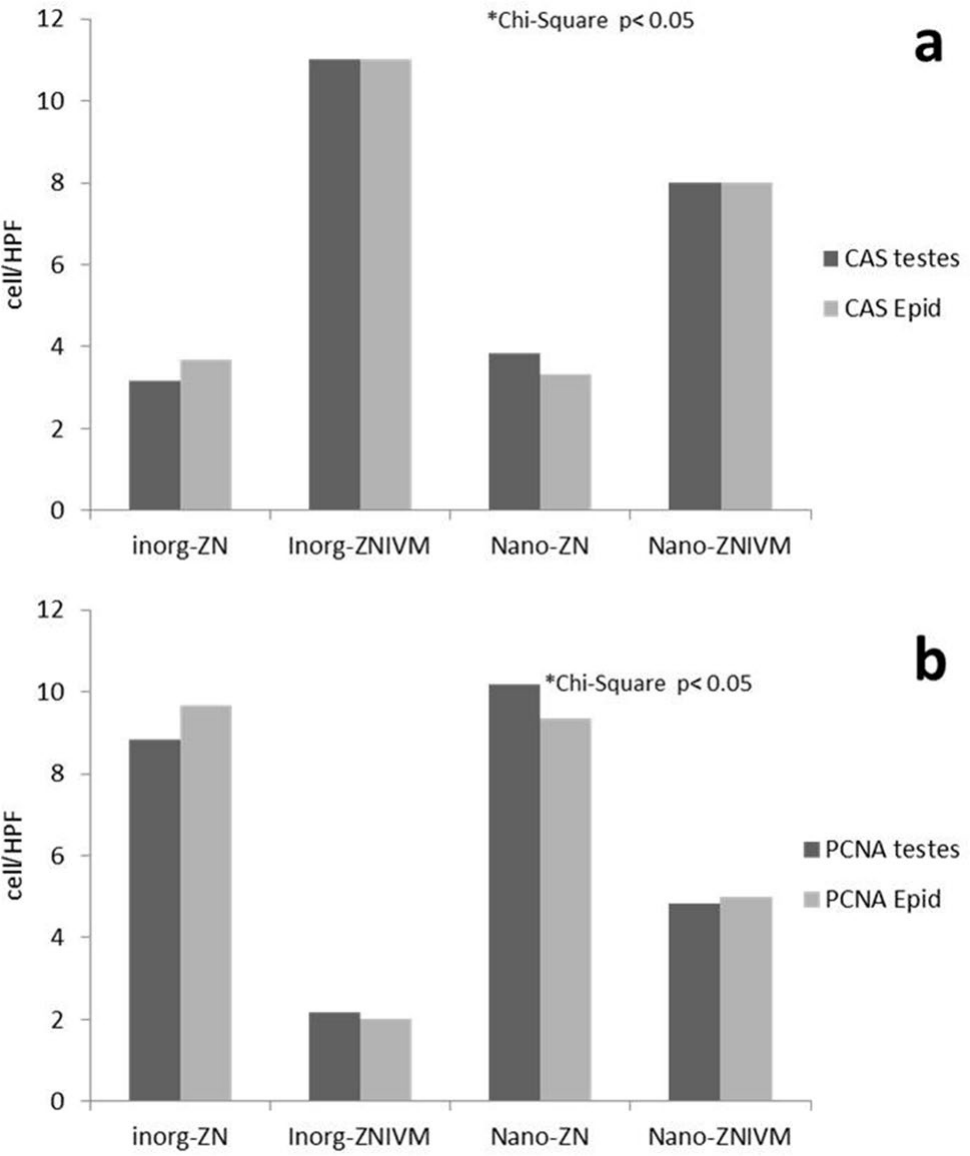
Histomorphometric analysis of immune expression of **a** caspase-3 and **b** PCNA in testicular and epididymal tissues of rabbits; all scores were subjected to non-parametric analysis using Kruskal–Wallis test to assess the significance between mean scores obtained from Wilcoxon rank-sum test (*P* > chi-square < 0.05)

On the contrary, as shown in Figs. 5 and 6, the proliferative activity of testicular and epididymal cells in the experimental groups was assessed using immunohistochemical localization of the proliferating cellular nuclear antigen (PCNA). In the testes (Fig. 6), the immunoreactivity of PCNA was mildly lower in the IVM and ZnNPs + IVM groups compared to the control groups (CTR and ZnNPs). Meanwhile, in the epididymal tubules, the proliferative activity was significantly decreased in the lining epithelium and increased in intraluminal tissue debris (Fig. 7). Figure 8 shows the non-parametric analysis for the mean count of immunological positive cells in testes and epididymis.

## Discussion

The present study found that the ZnNPs group that received nano-Zn replacement had the highest live BW, total body gain, and total feed intake and the best average feed conversion ratio compared to the control group that received inorganic Zn (CTR). Our results are consistent with those presented in Tag-El Din (2019), which showed that nano-Zn supplementation at 60 mg/kg improved the final BW, gain, FI, and FCR in growing rabbits. Consistent with this data, Hassan et al. (2017) reported that rabbits fed a diet supplemented with nano-Zn (30 and 60 mg/kg) had increased BW, gain, and FCR compared to the control group. Other reports revealed that the dietary supplementation of 60 mg/kg of nano-Zn/kg promoted the highest BW gain and better feed conversion ratio than the control in broilers (Zhao et al. 2014) or dual-purpose chickens (Siddhartha et al. 2016). These results can be explained by the smaller size of nano-Zn, which allows for easier and faster passage through the cell membrane, thereby increasing its bioavailability and efficiency in promoting proper physiological functions, including DNA and protein syntheses, leading to better growth (Case and Carlson 2002; Onuegbu et al. 2018). Zn also participates in several enzymatic and metabolic functions, including carbohydrate, lipid, and protein metabolism (MacDonald 2000; Prasad and Kucuk 2002). Moreover, Zn is essential for free radical scavenging, immune system enhancement, and protection of the pancreatic tissue against oxidative damage, thereby ensuring optimal pancreatic function in secreting the digestive enzymes and subsequently improving nutrient digestibility (Zhao et al. 2014; Saleh et al. 2018).

On the contrary, our findings revealed that weekly IVM injections significantly deteriorated the growth and feed intake among rabbits receiving dietary inorganic Zn. Similarly, El-Shobokshy et al. (2022) found that repeated injection of IVM in female rabbits caused a decrease in BW, TBWG, and TFI. Similar to our findings, Khaldoun-Oularbi et al. (2015) and Khaldoun Oularbi et al. (2017) studied the adverse effects of emamectin benzoate in rats and concluded that the final BW and BWG decreased significantly due to a significant decrease in FI caused by the loss of appetite with decreasing in gastrointestinal tract nutrient absorption and deterioration of food conversion efficiency (Ball and Chhabra 1981; Sheriff et al. 2002). Our results also agree with those of Chahrazed et al. (2020) who observed that the repeated injection of high doses of IVM (2 mg/kg BW subcutaneously, 3 doses per week) for three consecutive weeks in rabbits significantly (*P* < 0.05) decreased the TFI due to the decreased in appetite, which significantly decreased the BW and total gain. Fortunately, the present study revealed that replacement of inorganic Zn with nano-Zn could overcome the negative effects of repeated IVM injections on the growth and FI. Hence, the co-administration of nano-Zn had ameliorative effects by reversing the negative effects of IVM on the BW, gain, and feed consumption in rabbits.

The present study revealed that nano-Zn significantly enhanced the dressing percentage and had no significant effect on the relative weights of the skin, head, and spleen. Meanwhile, rabbits receiving nano-Zn had significantly lower (*P* < 0.0001) relative weights of the heart, lungs, kidneys, and livers compared to those receiving inorganic Zn (CTR). Consistent with our results, Tag-El Din (2019) found that supplementation with nano-Zn at 60 mg/kg non-significantly decreased the relative weight of the head; however, they found that nano-Zn had no significant effect on the kidney, liver, and heart percentage. Similar to our findings, El-Katcha et al. (2017) reported that nano-Zn (45 and 60 mg/kg) improved the dressing percentage, lowered the relative weight of the liver, and had no significant effect on the relative weight of the spleen in broiler chickens compared to dietary inorganic Zn. Moreover, Lina et al. (2009) stated that nano-Zn significantly elevated the dressing percentage of boiler chickens.

Our data clarified that repeated IVM injections caused the worst dressing percentage and organ relative weights, whereas dietary nano-Zn (*P* < 0.0001) improved the dressing percentage and reversed the negative effects of IVM injection on organ relative weights, especially of the spleen and liver. Similarly, previous studies by Khaldoun-Oularbi et al. (2013), El Zoghby et al. (2015), and Khaldoun Oularbi et al. (2017) revealed that IVM significantly increased rat liver weight, whereas IVM co-treatment with, for instance, vitamin C decreased the absolute and relative weight of the liver. Moreover, Chahrazed et al. (2020) found that repeated high-dose injections of IVM in rabbits increased the relative weight of the liver and decreased lung weight due to IVM accumulation in lung tissue and generation of oxidative stress due to continuous IVM injection (Al-Jassim et al. 2016), whereas vitamin C reversed the aforementioned changes and protected the weights of the internal organs from the negative effects of IVM hazards.

Testosterone is a steroid hormone from the androgen group in mammals, reptiles, birds, and other vertebrates. In mammals, testosterone is primarily secreted in the testicles of males and the ovaries of females, although small amounts are also secreted by the adrenal glands (Vodo et al. 2013; El-Far 2013). Free testosterone, the serum testosterone not bound to sex hormone-binding globulin or albumin, is biologically active and able to exert its effects by permeating into cells and activating its receptor (Kevin et al. 2012; El-Far 2013). From the obtained data, we can clearly observe a significant increase in serum testosterone in the ZnNPs group at 16 weeks old, which may have been due to the role Zn plays in several biochemical processes and physiological functions. Reports have shown that Zn is required for the normal function of numerous structural proteins, enzymes, and hormones necessary for growth and development (Bao et al. 2009; Abdel-Wareth et al. 2020). The improved concentrations of testosterone in response to nano-Zn replacement might be due to the increased number of Leydig cells in the testis, which increases testosterone production (El-Masry et al. 1994; Imam et al. 2009; Abdel-Wareth et al. 2020). At 18 weeks old, no significant difference in testosterone concentration was observed between the ZnNPs and ZnNPs + IVM groups, with both groups reaching puberty at the same age and earlier than the other groups. These results highlight the vital role of ZnNPs as a powerful antioxidant that stimulates the process of steroid genesis and release of GnRH hormones from the anterior pituitary gland. Moreover, Zn acts as a scavenger for excessive superoxide radicals, thereby exhibiting antioxidant-like activities (Gavella and Lipovac 1988; Baiomy et al 2018). Chia et al. (2000) suggested that Zn may bind with free radicals in the seminal plasma, produced by abnormal spermatozoa, thereby decreasing the concentration of this element. Zn deficiency causes a lowering in testosterone levels (Hadwan et al. 2012; Chia et al. 2000).

In line with this, the present study revealed that dietary nano-Zn replacement significantly enhanced Zn concentrations in the testes of ZnNP-treated group. Our results were consistent with those obtained by Zhao et al. (2014) who found that serum Zn concentrations were significantly higher in broilers receiving 60 or 100 mg/kg nano-Zn compared to those receiving inorganic Zn. Moreover, Hassan et al. (2017) found that nano-Zn supplemented groups had significantly greater hepatic and serum Zn contents (*P* < 0.001) compared to the inorganic Zn groups. Moreover, similar findings in Japanese quails were reported by Reda et al. (2020). Given the increased concentration of Zn in ZnNPs group and its antioxidant role, it was unsurprising that rabbits treated with ZnNPs showed the best oxidative status. Zn is a fundamental component in SOD and is involved in the cellular scavenging of free radicals and reactive oxygen species (Prasad 2008; Abd El-Hack et al. 2018). MDA is an important index for lipid peroxidation and oxidative damage caused by reactive oxygen species (Bin-Jumah et al. 2020; El-Far et al. 2020). Our results revealed that GSH, SOD, and CAT activities had significantly increased in the testes of the ZnNPs group, whereas MDA concentrations had decreased. The depressed MDA levels in our study resulted from the suppressive effects of dietary nano-Zn replacement on reactive oxygen species generation, thereby decreasing MDA levels. These results are consisted with that reported by Reda et al. (2021) who found that dietary nano-Zn level significantly (*P* < 0.001) enhanced serum SOD and glutathione peroxidase (GPX) but reduced MDA levels. Moreover, Kamel et al. (2020) stated that the addition of nano-Zn into the diets of rabbits significantly increased glutathione and superoxide dismutase activities and decreased MDA levels. Moreover, Tag-El Din (2019) reported that plasma CAT content was slightly elevated following high-dose nano-Zn supplementation (60 mg/kg) in the diet of growing rabbits.

In contrast, rabbits in the IVM groups showed lower enzymatic activity of GSH, SOD, and CAT and higher MDA levels. This result highlights the harmful and immunosuppressive effects of IVM injection in rabbits. Omshi et al. (2018) stated that repeated IVM administration was associated with oxidative degradation in male rats. GabAllh et al. (2017) and Al-Jassim et al. (2015) also reported that IVM injection adversely affects the immune status of rabbits. However, the present study showed that IVM injection slightly affected the nano-Zn supplemented group. This result suggests the potential role of nano-Zn supplementation in alleviating the stressful effects of IVM injection. Such findings could be attributed to the enhanced Zn concentration resulting from nano-Zn supplementation, which plays a vital role in inhibiting seminal oxidative stress (Marzec-Wróblewska et al. 2012). These results are in agreement with those of Kamel et al. (2020) who reported that dietary nano-Zn supplementation improved the immune status of heat-stressed rabbits. Moreover, Saleh et al. (2018) reported similar findings in heat-stressed birds.

The effect of IVM on genital organ dimensions was demonstrated in the IVM group, especially with regard to the size and weight of the testis plus epididymis and weight of accessory glands. This result was in agreement with that reported by El-Far (2013) who demonstrated that therapeutic and double therapeutic doses of IVM in male rats significantly decreased testes size. The mentioned study attributed these changes to the degenerative changes observed in double therapeutic dose of IVM associated with complete necrosis and complete absence of spermatogenesis in most seminiferous tubules. The epididymis showed degeneration and necrosis in the epithelial cells of the epididymal tubules. These results are supported by our histopathological findings, which revealed severe testicular damages in the form of abnormal spermatogenic cell arrangement, focal degenerative and necrotic changes, and cellular debris occupying the tubular lumen in groups treated with IVM. Similar results have been previously reported by several authors (Elzoghby et al. 2015; Ahmed et al. 2020). Similarly, GabAllh et al. (2017) noticed degeneration and vacuolation of spermatogenic cells at therapeutic doses of IVM after 4 and 8 weeks. These lesions were associated with necrosis of the epithelial cells lining the seminiferous tubules with pyknotic nuclei and formation of a few sperm giant cells at a therapeutic dose after 8 weeks. Meanwhile, the same degenerative changes were also observed at double therapeutic dose wherein complete necrosis and complete absence of spermatogenesis in most of seminiferous tubules with high number of sperm giant cells were noted. In the current study, immunohistochemical staining with PCNA revealed a reduction in testicular tissue immune expression among groups treated with IVM, with PCNA playing a pivotal role in the S-phase of the cell cycle via DNA synthesis and replication (Strzalka and Ziemienowicz 2011). Therefore, the reduction in PCNA expression may promote suppression of DNA synthesis with subsequent DNA damage and reduced cellularity and spermatogenesis (Moshari et al. 2017). Similar results were obtained by Ahmed et al. (2020). In the current study, the immunocytochemical staining of caspase-3 revealed strong immunoreactivity in testicular tissues of IVM-exposed rabbits. Meanwhile, a significant reduction in the number of caspase-3 immune positive cells was observed in rabbits with a ZnNP-supplemented diet. The excessive reactive oxygen species production from the mitochondria of testicular cells could potentially interfere with the proper release of cytochrome c, which subsequently activates caspase-3 and induces cell apoptosis (Simon et al. 2000; Chung et al. 2003). IVM had been reported to collapse the mitochondrial membrane, leading to cell death and activation of various apoptotic markers, including caspase-3 (Khafaga and El-Sayed 2018). Similar results were previously obtained by Ahmed et al. (2020).

IVM affected semen parameters, such that the IVM group had significantly lower semen motility, livability, and concentration than the other groups. This result was in agreement with those reported by Elzoghby et al. (2015) who found that therapeutic and double therapeutic doses of IVM in male rats significantly decreased total sperm count and mortality. The accumulation of free radicals has been associated with a significant decrease in sperm motility and sperm plasma membrane integrity and a significant increase in sperm abnormality and DNA damage, leading to infertility (Potts et al. 2000). ZnNP-treated group had significantly better semen parameters than the other groups given that Zn plays a pivotal role in sperm cell function, including lipid flexibility, cell membrane stabilization (Chia et al. 2000), sperm capacitation, and acrosomal reaction (Eggert-Kruse et al. 2002). Moreover, reports had shown that Zn increased semen volume, total live sperm count, sperm motility, and conception in heat-stressed rabbits (El-Masry et al. 1994). Our findings clearly showed that ZnNPs + IVM group had better semen parameters than the IVM group, indicating the vital role of Zn in over 200 proteins and enzymes essential for male fertility (Kumar et al. 2006). Research has shown that Zn supplementation enhances the physical characteristics of semen, including ejaculate volume, sperm count, motility, seminal plasma antioxidants, and fertility rate (Amen and Muhammad 2016; Rahman et al. 2014; El-Speiy and El-Hanoun 2013; Rafique et al. 2010; Ghasemi et al. 2009; Oliveira et al. 2004; Maldjian et al. 1998). Rats treated with Zn showed an increase in sperm count, sperm motility, and testosterone levels, as well as improved testicular structure and spermatogenesis abnormalities caused by obesity (Ma et al. 2020). However, other studies have suggested no significant association between Zn and sperm quality (Eggert-Kruse et al. 2002; Lin et al. 2000).

## Conclusion

Replacement of inorganic Zn with Zn nanoparticles enhanced the BW, weight gain, and FCR of male rabbits. In addition, fertility and oxidative parameters, as well as histopathologic findings, were also improved in ZnNP-treated rabbits. Dietary supplementation of ZnNPs for IVM-intoxicated rabbit ameliorated the negative impact of IVM and improved the performance of male rabbits potentially via its antioxidant and antiapoptotic pathways. Hence, we recommend including ZnNPs in the diets of rabbit exposed to IVM injections.

## Data Availability

The data used to support the findings of this study are included within the article and the coding of the data is available from the corresponding author upon reasonable request.
